# Service of Professor N. S. Davis in Mercy Hospital

**Published:** 1872-01-15

**Authors:** 


### MERCY HOSPITAL SERVICE OF PROF. N. S. DAVIS.

SYPHILITIC RHEUMATISM AND DIPHTHERIA.

*Case* 1. This man complained of those symptoms which the books call syphilitic rheumatism; he had pericranial thickening over the eyebrow and the characteristic pains, and was put on the following medicine:

| | | |
|---|---|---|
| ℞ | Iodide Soda | ʒ iij. |
| | Hydrarg Bi-chloride | gr. j. |
| | Tinct. Stramonii | ℥ ss. |
| | Syr. Simp. et Aqua | ℥ jv. |

One teaspoonful three times a day.

In two or three days his mouth became sore, breath bad, glands about mouth painful, and the first impression was that the patient was unusually susceptible to mercury, and had been salivated. The medicine was substituted by Chlorate of Potash and Belladonna, and a weak solution of Carbolic Acid was given him to hold in his mouth at his pleasure. The inflammation extended over the whole surface back to the fauces, and in three days a diphtheritic membrane, thick as a wafer, covered the whole surface. Soon as it appeared he was ordered lactate of iron gr. j in a teaspoonful of water every two hours. This acts both locally and internally. He has continued this and the carbolic acid wash for a week, with rapid improvement. Great efficacy has been claimed for lactic acid in removing the membrane of diphtheria and membranous croup, by inhaling it from a solution. It disintegrates the diphtheritic coating better than any other agent the lecturer has used.

INTESTINAL RHEUMATISM.

*Case* 2. This man was admitted to the hospital this morning, and says he was taken three days ago with a violent pain or cramp in the bowels. The left side of his abdomen is tender under pressure, and at the ilio-cæcal junction there is tenderness and a little hardness. He was at one time sick at the stomach, and vomited. His symptoms plainly indicate some degree of peritoneal inflammation, but it is of slight extent for his abdomen is not full, tense and painful as in severe peritonitis. There are two conditions which often give rise to symptoms such as we have observed. First, a slight inflammation of the peritoneal coat with a lodgement of fecal material at the ilio-cæcal junction, causing tenderness and hardness, with obstruction and vomiting, which sometimes extends over the whole peritoneum, and ends fatally in two or three days. Another condition is observed—especially in extreme cold weather. The attack commences with severe abdominal pains, tenderness, but little hardness, and no bloating. The tongue is clean, but the patient feels that tension of the abdomen which makes him think physic would give relief. Yet cathartics invariably aggravate the symptoms. This condition generally depends on true rheumatic inflammation of the muscular coat of the intestines, and sometimes becomes very protracted.

A number of these cases occur every winter, and the patient before us appears to be one of this class. The inflammation chiefly affects the colon, and extends to the peritoneum slightly. Considering the pain caused by movements in other parts affected with rheumatism it is easy to see how the peristaltic motions of the bowels should cause severe pain, particularly during the action of a cathartic.

The treatment consists mainly in the use of anodynes and alkaline salts. It is best to choose such medicines as will act freely on the skin and kidneys. The patient was ordered the bi-carbonate of soda and acetate of potash, each gr. x, to be taken every four hours, and alternating with Dover's powder gr. viij, nit. potassa v grs. and hydg. chl. mite gr. ij—the mild chloride to be omitted after the first twenty-four hours. Relief may be facilitated by narcotic fomentations applied to the abdomen; in family practice fomentations of hops may be used, or the infusion of hops may be used to make linseed meal poultices large enough to cover the painful part of the abdomen.

---

THE APPLICATION OF FORCEPS.— George Johnston, M. D., Master of the Rotunda Lying-in-Hospital, in his report for 1870, states that in 1,087 cases of labor the forceps was resorted to 83 times.

